# Pathogenic Prion Protein Isoforms Are Not Present in Cerebral Organoids Generated from Asymptomatic Donors Carrying the E200K Mutation Associated with Familial Prion Disease

**DOI:** 10.3390/pathogens9060482

**Published:** 2020-06-18

**Authors:** Simote T. Foliaki, Bradley R. Groveman, Jue Yuan, Ryan Walters, Shulin Zhang, Paul Tesar, Wenquan Zou, Cathryn L. Haigh

**Affiliations:** 1 Laboratory of Persistent Viral Diseases, National Institute of Allergy and Infectious Diseases, Division of Intramural Research, Rocky Mountain Laboratories, National Institutes of Health, 903 South 4th Street, Hamilton, MT 59840, USA; simote.foliaki@nih.gov (S.T.F.); bradley.groveman@nih.gov (B.R.G.); ryan.walters@nih.gov (R.W.); 2 Departments of Pathology and Neurology, Case Western Reserve University School of Medicine, Cleveland, OH 44106, USA; jue.yuan@case.edu (J.Y.); shulin.zhang@uky.edu (S.Z.); wxz6@case.edu (W.Z.); 3 Department of Genetics and Genome Sciences, Case Western Reserve University School of Medicine, Cleveland, OH 44106, USA; paul.tesar@case.edu

**Keywords:** prion, CJD, E200K, cerebral organoid, iPSC

## Abstract

Cerebral organoids (COs) are a self-organizing three-dimensional brain tissue mimicking the human cerebral cortex. COs are a promising new system for modelling pathological features of neurological disorders, including prion diseases. COs expressing normal prion protein (PrPC) are susceptible to prion infection when exposed to the disease isoforms of PrP (PrPD). This causes the COs to develop aspects of prion disease pathology considered hallmarks of disease, including the production of detergent-insoluble, protease-resistant misfolded PrPD species capable of seeding the production of more misfolded species. To determine whether COs can model aspects of familial prion diseases, we produced COs from donor fibroblasts carrying the E200K mutation, the most common cause of human familial prion disease. The mature E200K COs were assessed for the hallmarks of prion disease. We found that up to 12 months post-differentiation, E200K COs harbored no PrPD as confirmed by the absence of detergent-insoluble, protease-resistant, and seeding-active PrP species. Our results suggest that the presence of the E200K mutation within the prion gene is insufficient to cause disease in neuronal tissue. Therefore, other factors, such as further genetic modifiers or aging processes, may influence the onset of misfolding.

## 1. Introduction

Prion diseases are rare transmissible neurodegenerative diseases that are invariably fatal. These diseases affect both humans and animals. Creutzfeldt–Jakob disease (CJD) is the most common form of human prion disease, with sporadic CJD (sCJD), having no known cause, accounting for ~85–90% of prion disease cases. Familial CJD, caused by mutations within the PrP gene, and acquired CJD, caused by exposure to prion-infected materials, are less common types of CJD, accounting for ~10–15% and <1% of human prion diseases, respectively [1]. Familial and acquired CJD are the most studied because they have known causes, which can be modelled in experimental systems.

The hallmark features of prion diseases include deposits of insoluble, protease-resistant prion protein isoforms (PrPD) through a conformational transition of its cellular form (PrPC) accompanied by the presence of vacuoles, producing a spongiform appearance, in the brain. The PrPD within a prion disease brain can ‘seed’ formation of further PrPD by templated protein misfolding. Animal and cell culture models have been generated that demonstrate many of the features of human disease. These models have provided substantial insights into the pathogenesis of these disorders, including how the disease hallmarks including PrPD species and grey matter spongiform pathology, which are most evident in humans at terminal disease stage, evolve during the disease progression. In animal models of acquired prion disease, protease-resistant PrPD are detected in the brain considerably before the clinical stage, and coincide with development of some pathological features such as spongiosis and astrogliosis [2,3]. Seeding activity by PrPD can be detected before protease-resistant PrPD can be distinguished and prior to evidence of pathological changes. However, distribution of PrPD seeding activity in the brains of mice does not necessarily correlate with neurodegeneration [4] and the onset of neuropathology does not always correlate with the detection of PK-resistant PrPD [5].

Numerous mutations are linked with familial prion disease. E200K CJD is the most common familial CJD with ~98% penetrance, where a glutamic acid at codon 200 of PrP gene (*PRNP*) is substituted with a lysine. Mouse models of E200K CJD develop normally and their propensity to develop spontaneous disease appears dependent upon the genetic background of the mouse [5,6]. In mice that do develop disease, neurological symptoms onset at approximately 5–6 months old and mice progress to death at approximately 12–14 months old. Prion pathology, similar to human E200K CJD, is detected in the brains of these mice [5]. In a different model of E200K *PRNP* mutation, mice do not develop disease and live for more than 850 days [6]. Together, these data suggest that the presence of the E200K mutation alone is insufficient to cause disease and is influenced by host genetics.

Recently, it has been shown that human cerebral organoid (CO) cultures can propagate sCJD prions [7]. COs are three-dimensional, self-organizing spheres of neuronal lineage cells, which can be generated from induced pluripotent stem cells (iPSCs). The use of iPSCs allows for generation of COs from any donor, including those that are carriers of the *PRNP* E200K mutation. The first study generating E200K COs found no amyloid pathology up to 110 days in vitro. However, the authors did not specifically consider PrP deposition/pathology [8]. At 110 days old, it is unlikely that prion disease pathology would be observed because the COs are still maturing, and it is not until approximately 140 days old that astrocytes can be detected in 100% of COs [9]. Astrocytes are thought to play an important role in prion diseases and astrocytosis can be found within the brain of patients with prion disease [10]. Therefore, in similarity with the animal models of E200K CJD, young E200K COs may show no prion pathology but these features might develop with aging.

To investigate the influence of the *PRNP* E200K mutation on the production of PrPD in human brain tissue, we used iPSCs from donors carrying the E200K mutation to differentiate human COs. Organoids generated from two different donors showed no signs of PrP seeding activity, protease-resistant PrP, insoluble PrP or significant changes in their overall health for up to twelve months in culture.

## 2. Results

### 2.1. E200K Organoid Viability

**Figure 1:**
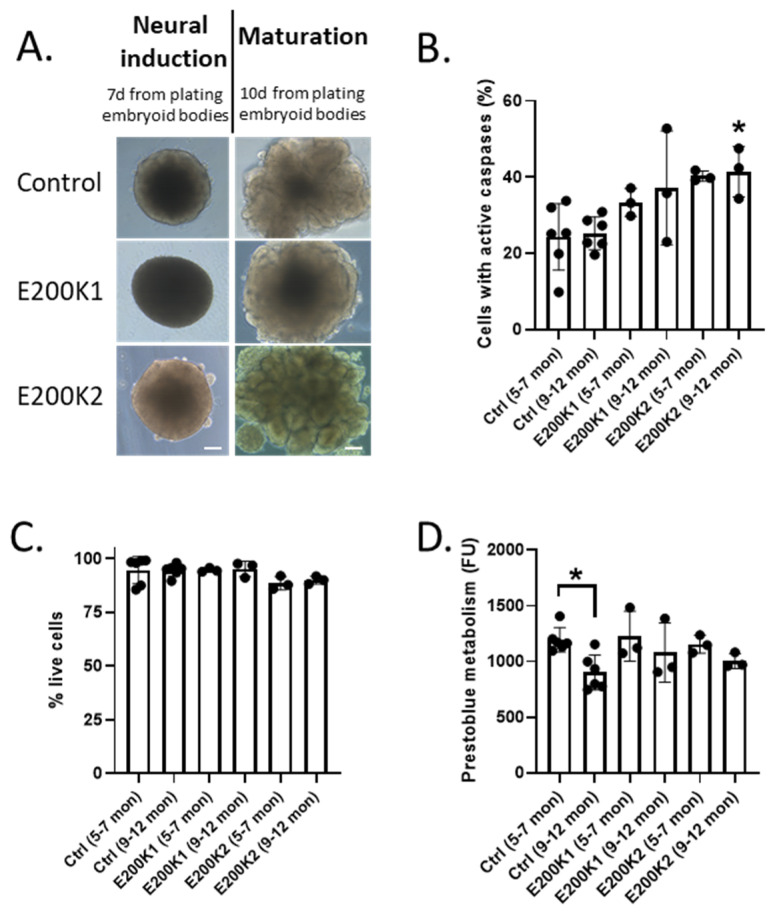
Cell viability of mature organoids. (**A**) Example images of control and E200K organoids. Magnification 10×; scale bar = 50 µm. (**B**) Percentage of active total caspases in negative control (*n* = 6; Ctrl), E200K1 (*n* = 3), and E200K2 (*n* = 3) organoids at 5–7 and 9–12 months post-differentiation. (**C**) Percentage of live cells in negative control (*n* = 6; Ctrl), E200K1 (*n* = 3), and E200K2 (*n* = 3) organoids at 5–7 and 9–12 months post-differentiation. (**D**) Prestoblue cell viability of live cells in negative control (*n* = 6; Ctrl), E200K1 (*n* = 3), and E200K2 (*n* = 3) organoids at 5–7 and 9–12 months post-differentiation. Negative controls contain data pooled from COs generated from two donors without any prion disease-related mutation. * *p* < 0.05, one-way ANOVA.

Recent studies have shown that COs increase in cellular complexity and maturity as they age. By 5 months post-differentiation (mpd) most cell types are fully differentiated and organoids have reached near maximum cellularity [9,11]. Neuronal function continues to develop with increasing activity measured to 8 mpd [11]. Hence, here, we studied two age groups of mature COs, 5–7 and 9–12 mpd. We utilized two control lines of COs (without any prion disease-related mutation) and two lines from two donors (1 male aged 50 and 1 female aged 32) carrying the E200K mutation (codon 129 MM), referred to herein as E200K1 and E200K2 (sequencing data are shown in Supplementary Figure S1). Both E200K donors were asymptomatic at the time of skin punch biopsy. Since no significant differences were discernible between the control lines (data not shown), these results are shown combined for simplicity. No morphological difference was seen between the control and E200K organoids during differentiation, with all forming COs of similar appearance (Figure 1A). To determine whether there was any significant age or E200K mutation-related cell death, we measured the number of cells with active caspases, a cellular correlate of apoptotic cell death. We found that within an organoid line, there were no age-related differences (Figure 1B). Relative to the age-matched negative control, only E200K2 COs contained significantly more cells with active caspases at 9–12 months (Figure 1B). Caspase activation is, however, also linked with cellular functions unrelated to death [12], and therefore could offer an over-representation of dying cells. To measure the number of live cells, we used a membrane impermeable dye, which is excluded from live cells but fluoresces within dead cells. Regardless of the age of the COs, the live cell count showed no significant difference in the percentage of live cells between the negative controls and E200K COs (Figure 1C). To further look at cell viability, a prestoblue cell viability assay, which measures cell metabolism, was performed. The prestoblue metabolism confirmed no significant differences between the metabolism of the age-matched controls and the E200K COs. A small age-dependent reduction in metabolism at 9–12 months old was significant in the control COs (Figure 1D). These data reveal that E200K mutation has little or no effect on the number of dying cells within the organoids. 

### 2.2. Expression of PrP Is Different between the Two Lines of E200K Organoids

**Figure 2:**
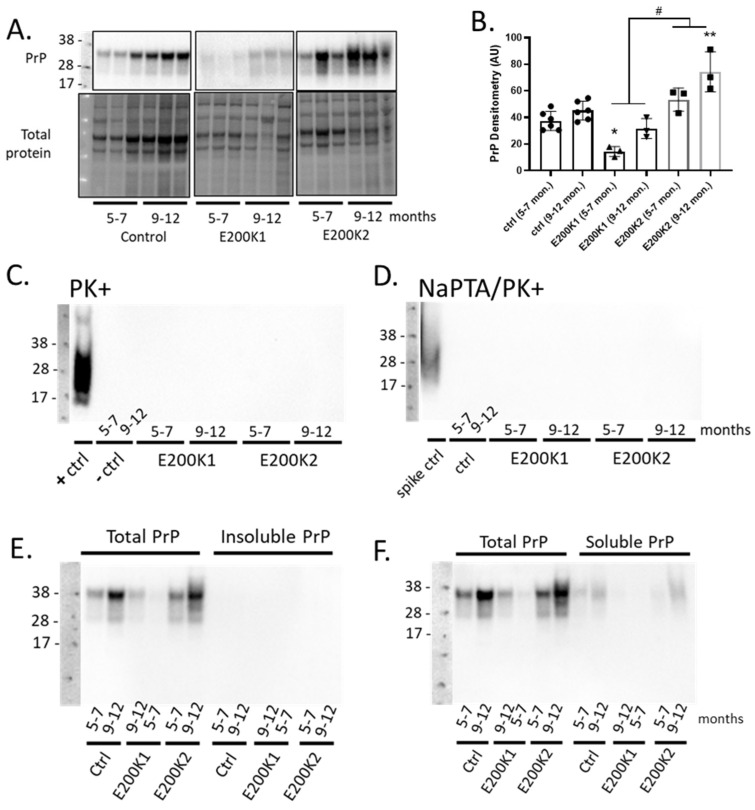
No detectable detergent-insoluble and PK-resistant PrP are present in E200K organoids. (**A**). Western blotting for total PrP in the negative control (Ctrl; top left panel; *n* = 3), E200K1 (top middle panel; *n* = 3 per an age group), and E200K2 (top right panel; *n* = 3 per an age group) organoids at 5–7 and 9–12 months after differentiation. The corresponding lower panels are the loading controls showing total proteins loaded per lane. (**B**) Densitometry quantification of the blots in A corrected for protein loading. * *p* < 0.05, ** *p* < 0.01 from the age-matched control, # *p* < 0.05 E200K1 versus E200K2. (**C**) Western blotting analysis for PrP following PK digest of lysates from control organoids known to be positive (+ctrl; *n* = 1) and negative (−ctrl; *n* = 1 per age group) for PK-resistant PrP, compared to 5–7- and 9–12-month-old E200K1 (*n* = 3 per age group) and E200K2 (*n* = 3 per age group) organoids. (**D**) Western blotting analysis for PK-resistant PrP following sodium phosphotungstic acid (NaPTA) of lysates spiked with PK-resistant PrP, as well as lysates from 5–7- and 9–12-month-old negative control (ctrl), E200K1, and E200K2 organoids. (**E**,**F**) Western blotting analysis for how much sarkosyl insoluble PrP (**E**) and soluble PrP (**F**) in lysates of 5–7- and 9–12-month-old negative control (ctrl), E200K1, and E200K2 organoids as compared with the total PrP level.

The expression level of PrP is usually significantly increased during the clinical stages of mouse models of acquired prion diseases [2,13]. In transgenic mice expressing chimeric mouse/human PrP with the E200K mutation, the PrP protein level at the end stage of the disease was significantly increased as indicated by the increased PrP immunoreactivity [5]. To determine any age-dependent change of total PrP expression in E200K organoids, we immunoblotted for total PrP in the E200K COs relative to the negative controls at 5–7 months and at 9–12 mpd. Significantly more PrP was detected in the control and E200K1 COs at 9–12 mpd relative to 5–7 mpd. The E200K2 COs appeared to have a similar age-dependent increase in PrP expression but this was not statistically significant (Figure 2A,B). Relative to the age-matched negative controls, E200K1 COs expressed lower total PrP, which was significantly lower at 5–7 mpd. The E200K2 COs expressed higher total PrP than the E200K1 COs, and this was significantly different from the control PrP expression levels at 9–12 mpd (Figure 2B).

### 2.3. E200K COs Do Not Harbor Insoluble and PK-Resistant PrPD

Proteinase K (PK)-resistant PrPD (PrPres) species are detectable in brains of patients who died from familial CJD caused by E200K mutation [14]. PK-resistant PrPD species are also detectable in brains of the mouse model of E200K mutation at the terminal stage of the disease [5]. To determine whether the E200K COs generated PK-resistant PrPD, we immunoblotted for PrP in lysates from E200K COs and aged-matched negative controls following treatment with PK. We used a lysate from COs infected with sporadic CJD, as published previously [7], for our positive control. We found that regardless of the age, there was no detectable PrPres in both the negative controls and E200K COs as compared to a substantial level detected in the positive control (Figure 2C). In addition, NaPTA precipitation of these samples prior to the PK digest and PrP immunoblotting, a technique used to enhance the detection of minimal levels of PrPres, confirmed no PrPres in the E200K COs like the negative controls (Figure 2D).

Given that misfolded and abnormal PrP can be PK sensitive despite being insoluble, we utilized a sarkosyl insolubility assay to determine the levels of insoluble/soluble PrP in the E200K COs as compared to the age-matched negative controls. We found that irrespective of the age, E200K COs exhibited no insoluble PrP (Figure 2E). Detectable levels of soluble PrP were evident in these COs, albeit substantially lower than the total PrP levels (i.e., before the insolubility assay; Figure 2F).

### 2.4. No PrPD Seeding Activity in E200k Organoid Up to 12 Months Old

**Figure 3:**
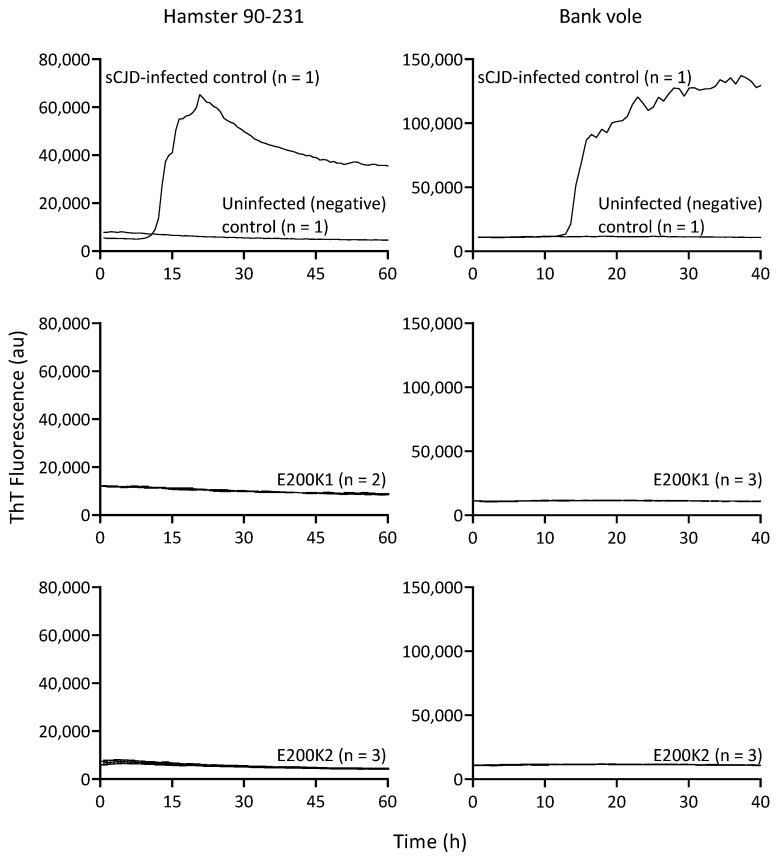
Seeding activity of PrP in E200K organoids. RT-QuIC analysis was performed on 12-month-old E200K1 (*n* = 2; **middle** panel) and E200K2 (*n* = 3; **bottom** panel) organoids. Reactions were run in quadruplicate for each organoid using hamster recombinant PrP90–231 (**left**) or bank vole recombinant PrP23–230M109I (**right**). The data are displayed as the mean from all the organoids tested in each group. Uninfected and sCJD infected organoids were run as controls (**top** panel).

One significant biophysiological correlate of pathogenic E200K PrPD is the ability to seed the propagation of more PrPD in vitro [15,16]. To determine whether there was any pathogenic PrP in the E200K COs that might be too low to be detected by immunoblot analysis, we assessed the ability of PrP in the E200K COs to seed propagation of PrP aggregates utilizing a highly sensitive technique called RT-QuIC [17,18,19]. Samples were tested using both hamster PrP90–231 and bank vole PrP20–230 as substrates for seeding. The hamster 90–231 PrP substrate has been shown to detect PrPD seeding activity with very high sensitivity [20,21]. However, some strains of PrPD do not react with hamster 90–231, but all known strains are detected by the bank vole 20–230 substrate [22]. We found that in contrast to the positive control from COs infected with sCJD and analogous to the uninfected negative control, both lines of E200K COs showed no seeding activity at a few months old up to 12 months old with either hamster 90–231 or bank vole 23–230 (Figure 3 and Table 1). These results confirmed that the E200K COs harbored no detectable PrP species capable of seeding the RT-QuIC assay.

## 3. Discussion

Herein, we found that COs produced from donor fibroblasts with the E200K mutation, associated with a high penetrance familial form of CJD, did not inherently develop disease-associated features. No prion seeding activity or disease-related PrP (proteinase K-resistant or insoluble PrP) were observed up to 12 mpd, which is a mature age range of COs. The typically increased detection of total PrP associated with the clinical stage of prion disease was not significantly evident in these E200K COs. Overall, CO health also seemed mostly unaffected by the presence of the mutation.

COs harboring the E200K mutation were previously used as negative controls for amyloid and Tau pathology in a study that demonstrated organoids from donors with Down Syndrome and familial Alzheimer’s disease spontaneously develop the pathological features of these disorders [8]. Mutations causally linked with hereditary AD and Down Syndrome likely produce increased steady-state levels of disease-associated amyloid-beta throughout life, with aggregation and deposition occurring once a threshold is reached. However, mutations within *PRNP* are not thought to produce a steady-state increase in disease-association prions—instead, it is believed that production starts later in life, maybe due to a triggering event. Our organoid model confirms that production of PrPD species is not innate within the E200K COs.

It remains unclear whether the known limitations of COs such as the lack of epithelial cells and microglia as well as the absence of vascular system [7] partly contributed to the absence of PrPD in the CO model. It is additionally conceivable that one-year-old E200K COs, while considered old in the life of an organoid, do not equate to the age at which PrPD is produced in vivo. We reason that since E200K COs were freshly differentiated from progenitor cells, even the one-year-old cultures might be too young to produce PrPD. Alternatively, like the E200K mice that live to old age disease free [6], these organoids may never develop E200K prion pathology in the absence of additional factors.

The difference in the detected level of total PrP between age-matched E200K1 and E200K2, despite harboring the same disease-causing mutation, suggests that the expression of PrP varies substantially between individuals. This is supported by previous studies, which showed wide variation of PrP expression in healthy control brains [23]. This may explain why some carriers of the E200K mutation do not develop the disease as well as why the clinical signs [24], age of clinical onset, and duration of clinical disease vary between individuals [25].

Further genetic modifiers may also influence disease onset, duration and signs. For instance, CYP4X1 gene has been recently found to modulate the age of onset in individuals with the E200K mutation [25]. The influence of genetic background on E200K prion disease is also shown by the difference in the outcomes of the two mouse models of E200K prion disease. The first transgenic mouse model engineered to express E200K PrP, where the E200K mutation was introduced into human PrP, failed to spontaneously develop the disease [6]. The second model where the E200K mutation was introduced into a chimeric mouse/human PrP expressed in mice with either a PrP knockout or wild-type background spontaneously developed prion disease [5].

In the mouse model of E200K disease that showed pathology and death, PrPres deposition is only evident at the terminal disease stage, much later than the onset of other pathological features such spongiform formation. This suggests that PrPD was PK sensitive at earlier stages of the disease or that pathology can occur below detectable levels of PrPD. Further, neuronal progenitor cells harboring the E200K mutation do not have PrPres and the pups born with the mutation are healthy [5,26]. This supports the hypothesis that PrPD species do not exist in the E200K mice at early ages, but they are produced at later ages, possibly as PK-sensitive PrPD, and become mature as PrPres by the terminal stage of the disease. This speculation is consistent with the report that E200K PrP appears to undergo additional modifications that are age-dependent to produce the disease isoforms [27]. Further studies are under consideration to determine whether factors that can trigger the production of PrPD in the E200K COs can be identified.

The absence of PrPD in E200K COs is consistent with a previous finding that, in a cell culture model of E200K mutation, the mutant PrP exhibited similar biochemical properties to cellular prion protein [28]. Another study on a different hereditary prion disease mutation, Y218N, associated with Gerstmann–Straussler–Scheinker (GSS) syndrome, also supports that production of PrPD does not readily occur in vitro due to the presence of a PRNP mutation alone. In this study, the authors used iPSCs from a donor carrying the Y218N mutation to generate neuronal cells and spherical neural masses [29]. The Y218N cultures showed features typical of Y218N GSS in humans including astrogliosis, Tau hyperphosphorylation and increased cell death. However, the Y218N cultures did not produce PrPD. This suggests a functional deficit related to the disease-causing mutation, resulting in accumulation of phosphorylated tau in the absence of detectible PrPD.

This study addressed whether organoids from asymptomatic donors with the PRNP E200K mutation would develop an E200K-associated phenotype over a long period in culture. It has previously been shown that skin fibroblasts from symptomatic prion disease patients have RT-QuIC seeding activity [30,31] and that, in mice, seeding positivity occurs very early after infection [32]. Had we utilized fibroblasts from symptomatic patients, we may well have seen propagation within the organoids from pre-existing seeds. While we cannot rule out the possibility that prion seeds could not withstand and propagate throughout the re-programming and organoid differentiation process, this may provide an interesting opportunity to contrast organoids from symptomatic and asymptomatic donors.

## 4. Conclusions

Human COs offer a promising model for the study of familial neurological disorders, as they can be differentiated from donors carrying disease-causing genetic mutations and potentially manifest the associated pathology of that disorder. Herein, we produced COs using iPSCs from donors who carry the E200K mutation within the PrP gene, associated with a familial form of CJD with nearly complete penetrance. We found that mature E200K COs did not spontaneously generate the disease isoforms of PrP and did not demonstrate impaired survival in culture over long periods of time.

## 5. Materials and Methods

### 5.1. Human Ethics Statement

The human samples used in this study were obtained from commercial sources (ATCC) or from skin punch biopsy of asymptomatic donors carrying prion protein gene mutation at codon E200K (methionine homozygous at codon 129) at Case Western Reserve University following the Institutional Review Board (IRB) protocol IRB No. STUDY20181189 approved and monitored by University Hospitals Cleveland Medical Center and Case Western Reserve University School of Medicine. The informed consent form was signed and obtained from each donor. The skin punch biopsy samples were de-identified before being provided to the researchers at the NIH. Thus, the NIH Office of Human Subjects Research Protections (OHSRP) has determined these samples to be exempt from IRB review.

### 5.2. ATCC Human Induced Pluripotent Stem Cells (hiPSCs)

Two lines of hiPSCs, ATCC-HYS0103 (ATCC^®^ ACS-1020™) and KYOU-DXR0109B [201B7] (ATCC^®^ ACS-1023™), classified as ‘normal’ (no known neurodegenerative disease), were purchased from the American Type Culture Collection (ATCC).

### 5.3. Generation of E200K(1), E200K(2) and RAH019A hiPSCs

Donor fibroblasts were collected by skin punch biopsy and grown in DMEM supplemented with 10% (*v*/*v*) fetal bovine serum, 1× glutamax and penicillin/streptomycin. Reprogramming was achieved using ReproRNA™-OKSGM and ReproTeSR™ medium (Stemcell Technologies) as per the manufacturer’s instructions.

### 5.4. hiPSC Culture

Human iPSC lines were routinely cultured on low growth factor Matrigel (Roche) in mTeSR1 medium (Stem Cell Technologies) with 5% CO_2_ in a humidified incubator. Media were changed daily, and colonies were passaged at approximately 70–80% confluency before contact between colonies could occur, using ReLeSR (Stem Cell technologies) reagent as described in the manufacturer’s instructions.

### 5.5. Organoid Generation

Organoids were generated using the protocol described by Lancaster and Knoblich [33] or using the Cerebral Organoid Differentiation kit (Stem Cell Technologies), following the protocols as described using one T25 of iPSCs per 96-well plate.

### 5.6. Organoid Culture

Organoids were routinely cultured in complete maintenance medium (1× glutamax, 1× penicillin/streptomycin solution, 0.5× non-essential amino acids, 0.5% [*v*/*v*] N2, 1% (*v*/*v*) B12 with retinoic acid, 1 μL/4 mL insulin, and 1 μL/286 mL 2-Merceptoethanol in 1:1 Neurobasal:DME-F12 medium), under standard incubator conditions (5% CO_2_, 37 °C, humidified), on an orbital shaker at 85 rpm using vented conical flasks (Corning) as described previously [7].

### 5.7. Organoid Images

Organoid images were captured using a Leica DMIL LED inverted microscope with a Leica HC 170 HD digital camera.

### 5.8. RT-QuIC

Real-time quaking-induced conversion (RT-QuIC) assays were performed similarly to those reported previously [34]. Briefly, the RT-QuIC reaction mix contained a final concentration of 10 mM phosphate buffer (pH 7.4), 300 mM NaCl, 0.1 mg/mL hamster recombinant PrP 90–231 (purified as described in [34]) or bank vole recombinant PrP23–230 M109I [22], 10 μM thioflavin T (ThT), and 1 mM ethylenediaminetetraacetic acid tetrasodium salt (EDTA). Organoids were homogenized by motorized pestle to 10% (*w*/*v*) in PBS and cleared with a 2000× *g* 2 min centrifugation. Organoid homogenates were serially diluted in SDS/PBS/N2 solution, using 0.1% (*v*/*v*) for hamster PrP substrate or 0.05% (*v*/*v*) for bank vole, to a final SDS concentration in the reaction mix of 0.002% or 0.001% (*w*/*v*) respectively. A volume of 98 μL of reaction mix was loaded into a black 96-well plate with a clear bottom (Nunc), and reaction mixtures were seeded with 2 μL of a 103-fold dilution of organoid homogenate for a final reaction volume of 100 μL. Reactions were in quadruplicate for each sample. Plates were sealed (Nalgene Nunc International sealer) and incubated in a BMG FLUOstar Omega plate reader at 50 °C for 60 h with hamster or 42 °C for 40 h with bank vole substrate with cycles of 60 s of shaking (700 rpm, double-orbital) and 60 s of rest throughout the incubation. ThT fluorescence measurements (excitation, 450 ± 10 nm; emission, 480 ± 10 nm [bottom read]) were taken every 45 min. The CJD infectious control samples were handled in a dedicated BSL2 facility following established SOPs and RML biosafety guidelines.

### 5.9. Sodium Phosphotungstic Acid Precipitation

Sodium phosphotungstic acid (NaPTA) precipitation was performed as described previously [35]. Briefly, 1% (*w*/*v*) lysates were solubilized in sarkosyl for 10 min at 37 °C with constant agitation at 1400 rpm, digested with 25 U/uL Benzonase (in 2 mM MgCl_2_) at 37 °C for 30 min, incubated in 0.33% (*w*/*v*) NaPTA (containing 20 mM final concentration of MgCl_2_) at 37 °C for 30 min with constant agitation at 1400 rpm, and centrifuged at 14,000× *g* at RT for 30 min. A spike of 20 µL of 10% (*w*/*v*) CJD infected COs confirmed to contain PrPres into an uninfected lysate prior to the NaPTA precipitation was used as the positive control. The pellets were resuspended in 20 µL of 0.1% (*v*/*v*) sarkosyl, digested with 10 µg/mL PK for 1hr at 37 °C, boiled in 1× sample buffer (containing 2% [*v*/*v*] beta mercaptoethanol) for 5 min to stop the activity of PK, and analyzed by Western blotting, probing with 3F4 (1:10,000) anti-PrP antibody.

### 5.10. Detergent Insolubility Assay

We adapted the detergent insolubility assay from a previously published protocol [36]. Briefly, lysates (20 µL of 10% *w*/*v* in RIPA lysis buffer) were treated with 300 µL of 10% (*w*/*v*) sarkosyl for 1 h at RT with constant agitation at 1400 rpm, diluted with 2680 µL of H-Buffer, and centrifuged at 100,000× *g* for 1 h at 4 °C. The pellets were resuspended in 1× sample buffer for Western blot analysis. The proteins in the supernatants were precipitated by methanol precipitation as published previously [37] and resuspended in 1× sample buffer for Western blot analysis.

### 5.11. Active Multicaspase Assay

COs were dissociated into ~100 µL of single cells by an Accutase Cell Dissociation buffer (Thermofisher). For the positive control, COs were treated with 40% (*v*/*v*) ethanol for 10 min before being dissociated into single cells. Active caspases were then labelled with a fluorescent label for total caspases for 30 min at 37 °C and for cell death using 7-Aminoactinomycin D (7-AAD), a marker of loss of membrane integrity, using the Muse Multicaspase assay kit (Millipore-Sigma) as per the manufacturer’s instructions. The negative controls were cells without the label. A Muse Cell Analyzer was used to count cells with and without active caspases. Gates were set for caspase-negative (alive) and 7-AAD-positive (dead) using the positive ethanol-treated controls and negative (no dyes included/no fluorescence) controls.

### 5.12. Prestoblue Analysis

As published previously [7], COs were incubated in 10% (*v*/*v* in organoid media) Prestoblue reagent for 30 min at 37 °C. The fluorescence of the Prestoblue was measured in a ClarioStar plate reader (BMG) at excitation/emission wavelength of 560 nm/590 nm.

### 5.13. PK Digest and Western Blotting

Crude 10% (*w*/*v*) tissue lysates, before and after digestion with 10 µg/mL PK, as described previously [7], and the yields of NaPTA precipitation and Detergent insolubility assays were denatured by boiling for 5 min in 1× sample buffer (Thermofisher) containing 2% beta-mercaptoethanol. Denatured proteins were resolved in SDS-PAGE and transferred into a PVDF membrane for immunoblotting. The membrane was blocked with 5% (*w*/*v*) blocking solution (Thermofisher) for 1 h and PrP was probed with 3F4 antibody (Millipore) at 1:10,000 dilution, detected with anti-mouse HRP (Abcam), visualized by ECL Select (Amersham) and imaged on the iBright imaging system (Invitrogen). The total protein was visualized with a no-stain protein-labelling reagent (Invitrogen).

### 5.14. Data Analysis

Image J was used for the densitometry analysis. Statistical analysis was carried out in GraphPad Prism 7.04. If not stated otherwise, the levels of active caspases and PrP expression were compared between age-matched cell lines by one-way ANOVA on ranks with Dunn’s correction for multiple comparisons and between age groups by the unpaired Student’s *t* test.

## Figures and Tables

**Table 1:** RT-QuIC results from periodic testing over 1 year. Denominators show the number of individual organoids tested per time point/substrate. ND = not done.

Days Old	Substrate	RT-QuIC Positive	
		E200K1	E200K2
35	bank vole	0/3	ND
84	hamster 90–231	0/3	ND
112	hamster 90–231	0/3	ND
140	bank vole	0/3	ND
180	hamster 90–231	ND	0/3
>365	hamster 90–231	0/2	0/3
>365	bank vole	0/3	0/3

## Supplementary Materials

The following are available online at https://www.mdpi.com/2076-0817/9/6/482/s1.
