# Supplementary material for: Pathogenic Prion Protein Isoforms Are Not Present in Cerebral Organoids Generated from Asymptomatic Donors Carrying the E200K Mutation Associated with Familial Prion Disease
Source: Pathogens. 2020 Jun 18;9(6):482. doi: 10.3390/pathogens9060482 (PMC7350378; doi:10.3390/pathogens9060482)

Simote T Foliaki, Bradley R Groveman, Jue Yuan, Ryan Walters, Shulin Zhang, Paul Tesar, Wenquan Zou, Cathryn L Haigh. **Pathogenic prion protein isoforms are not present in cerebral organoids generated from asymptomatic donors carrying the E200K mutation associated with familial prion disease.**

Supplementary Figure 1. *E200K sequencing data.* Electropherogram data for donors with the E200K mutation also showing methionine homozygosity at codon 129 and the PrP reference sequence for comparison.

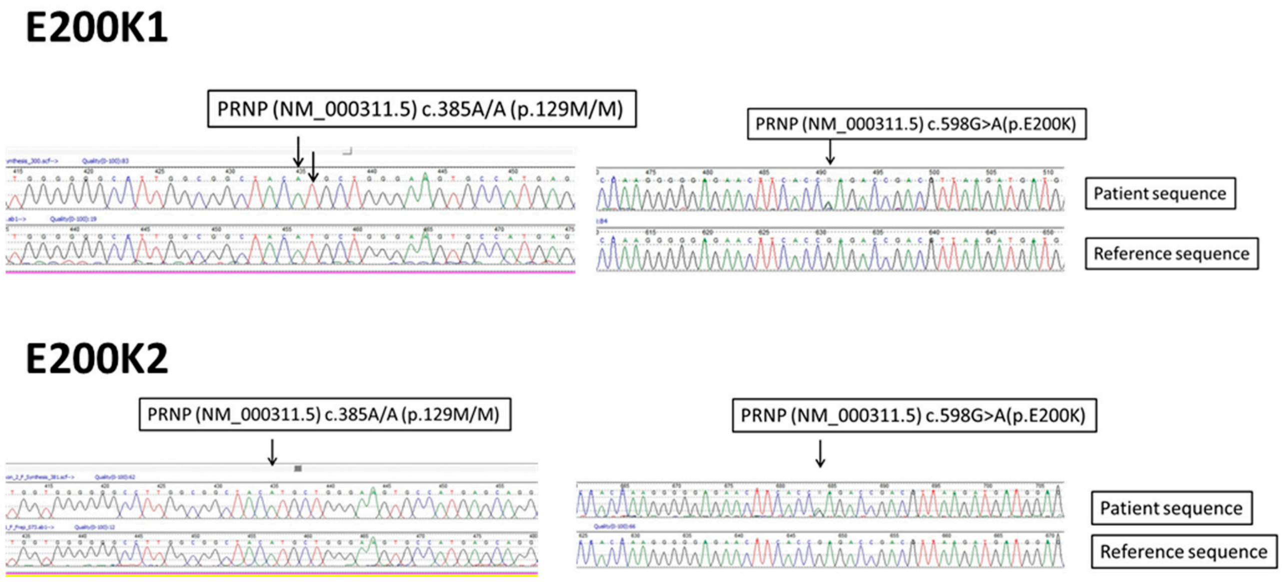

Supplement: Supplementary file 1 [file pathogens-09-00482-s001.pdf]
